# High dynamic range $B_{1}^{+}$ mapping for the evaluation of parallel transmit arrays

**DOI:** 10.1002/mrm.30349

**Published:** 2024-10-27

**Authors:** Jörg Felder, Markus Zimmermann, N. Jon Shah

**Affiliations:** ^1^ Institute of Neuroscience and Medicine – 4 Forschungszentrum Jülich Jülich Germany; ^2^ Faculty of Medicine RWTH Aachen University Aachen Germany; ^3^ Institute of Neuroscience and Medicine – 11 Forschungszentrum Jülich Jülich Germany; ^4^ JARA–BRAIN–Translational Medicine Aachen Germany; ^5^ Department of Neurology RWTH Aachen University Aachen Germany

**Keywords:** $B_{1}^{+}$ mapping, ESPIRiT, high SNR, ultrahigh‐field

## Abstract

**Purpose:**

Demonstration of a high dynamic‐range and high SNR method for acquiring absolute $B_{1}^{+}$ maps from a combination of gradient echo and actual‐flip‐angle measurements that is especially useful during the construction of parallel‐transmit arrays.

**Methods:**

Low flip angle gradient echo images, acquired when transmitting with each channel individually, are used to compute relative $B_{1}^{+}$ maps. Instead of computing these in a conventional manner, the equivalence of the problem to the ESPIRiT parallel image reconstruction method is used to compute $B_{1}^{+}$ maps with a higher SNR. Absolute maps are generated by calibration against a single actual flip‐angle acquisition when transmitting on all channels simultaneously.

**Results:**

Depending on the number of receiver channels and the location of the receive elements with respect to the subject being investigated, moderate to high gains in the SNR of the acquired $B_{1}^{+}$ maps can be achieved.

**Conclusions:**

The proposed method is especially suited for the acquisition of $B_{1}^{+}$ maps during the construction of transceiver arrays. Compared to the original method, maps with higher SNR can be computed without the need for additional measurements, and maps can also be generated using previously acquired data. Furthermore, easy adoption and fast estimation of receiver channels is possible because of existing highly optimized open‐source implementations of ESPIRiT, such as in the BART toolbox.

## 1 INTRODUCTION

With the introduction and increased application of ultrahigh‐field (UHF) imaging systems, parallel transmission (pTX) techniques have found wide interest. Because all pTX methods require the acquisition of 2D or 3D transmit sensitivity maps, interest in $B_{1}^{+}$ mapping sequences has also increased. Prominent examples are the double angle method,^1^ actual flip angle imaging (AFI),^2^ the Bloch‐Siegert shift method,^3^ the dual refocusing echo acquisition mode (DREAM) technique,^4^ and the presaturated turboFLASH method.^5^

Another driving factor for transmit field mapping has been the evaluation of novel transmit arrays where $B_{1}^{+}$ mapping is used to validate the simulation model by comparing simulated versus measured transmit field distributions. This step is essential as the simulation model is subsequently used to derive the specific absorption rate (SAR) properties of the array.^6^ Validation is conducted on phantoms. Generally, water‐based phantoms are used because they should provide a high SNR and a single resonance line.^6^ However, because of the complex interactions between the RF fields and the phantom at UHF, the maps acquired from phantoms usually show strong interference patterns that require high dynamic range field maps.

Because the dynamic range of many mapping sequences is not sufficient to depict $B_{1}^{+}$ maps of individual transmit elements of a pTX array,^7^ several solutions have been presented. One method proposes the acquisition of several maps when transmitting with multiple elements in an interferometric way and the subsequent decomposition of these maps into those for the individual coil elements.^8^, ^9^, ^10^ Another method, presented by van de Moortele et al.,^11^ used low flip‐angle gradient echoes to derive relative maps, which were then calibrated to absolute values using a single AFI acquisition that transmits on all channels. This method has been used to evaluate the field distribution of a number of pTX arrays, for example.^12^, ^13^, ^14^ However, the method shows low SNR and potentially requires signal averaging.^15^, ^16^ Similarly, Setesompop et al.^17^ used spoiled gradient echoes (SPGRs) whereby relative $B_{1}^{+}$ maps are acquired in combination with a single absolute $B_{1}^{+}$ map. This method has since been extended by Padormo et al.^18^ to include multiple RF drive levels for the SPGRs, which renders the acquisition of an absolute map obsolete.

In this work, we extend the method developed by van de Moortele et al.^11^ by replacing their post‐processing technique for estimating the relative transmit fields of individual coils with the ESPIRiT algorithm. Although originally designed to estimate receive coil sensitivities in parallel imaging, we show that ESPIRiT's mathematical framework is equally applicable to transmit fields. ESPIRiT uses an eigenvalue decomposition of the system matrix to distinguish between signal and noise, creating relative $B_{1}^{+}$ maps with enhanced SNR. This is achieved by isolating the dominant eigenvectors, which most accurately represent the true sensitivity profiles while suppressing components associated with noise. We further compare this approach to the method presented by Brunner et al.,^19^ which uses a voxelwise eigenvalue decomposition of the outer product of the transmit and receive channels to simultaneously determine the transmit and receive sensitivities. Although the method proposed by Brunner et al.^19^ also reduces noise by keeping only the dominant eigenvalues, our experiments demonstrate that ESPIRiT produces superior results, likely because of a more effective separation of signal and noise, possibly by exploiting a larger null space.

## 2 METHODS

In the original ESPIRiT algorithm, the receive field maps **C**_RX,*j*_ found in a SENSE‐like problem are estimated by

(1) $$P\;F\;C_{\mathrm{RX},j}\;x=d_{j},$$

with the underlying image, **x**, the sampling matrix, **P**, the discrete Fourier transform, **F**, the measured k‐space data, **d**, and the (receive) channel index, *j*, ranging from one to the total number of receive channels. It can be shown that a system matrix can be constructed by running a sliding window through a fully sampled auto calibration (AC) region of the k‐space data and that the sensitivity maps are those eigenvectors of the system matrix that correspond to the eigenvalues of one.

The problem of estimating the transmit field maps, **C**_TX,*I*_, from 2D complex (magnitude and phase), low flip‐angle and spoiled or fully relaxed gradient echo (GRE) images, acquired when transmitting with one channel at a time, can be rewritten in the same way. Given that the excited signal, **x**_*i*_, can be approximated as

(2) $$\sin\left(\alpha_{i}\right)M_{0}\approx \alpha_{i}M_{0}=x_{i},$$

with the flip angle, **α**_*i*_, given by **α**_*i*_ = γ $B_{1}^{+}$,_*i*_ T_RFE_, and $B_{1}^{+}$,_*i*_ being the transmit field of the (transmit) channel *i* within the range 1 to total number of transmit channels, γ is the gyromagnetic ratio, T_RFE_ is the hard pulse equivalent pulse duration, **M**_0_ the equilibrium magnetization, and **x**_*i*_ is the GRE image acquired when transmitting on channel *i*. By substituting the left‐hand side of Eq. (2) with the relative transmit field **C**_TX,*i*_, then applying a Fourier transformation on both sides and multiplying with a “sampling matrix” ***P*** = 1, one obtains.

(3) $$P\;F\;C_{\mathrm{TX},i}\;M_{0}=P\;F\;x_{i}=d_{i},$$

where **d**_*i*_ = **F x**_*i*_ is the Fourier transform of the recombined reconstructed image **x**_*i*_. It can be seen that Eqs. (1 and 3) are equivalent. Thereby, we assume that the transmit field maps, **C**_TX,*i*_, obtain features similar to those of the receive field maps, **C**_RX,*j*_, in terms of spatial smoothness.

In this formulation, the acquisition of uncombined GRE images is not required, because only combined images are required for map generation. The images are then projected back into k‐space by 2D Fourier transformation, and the k‐space data is used as input for the ESPIRiT algorithm. Assuming an array with a total number of *n* transmit elements and a matrix size of *n*_*x*_ × *n*_*y*_, the ESPIRiT algorithm returns a coefficient matrix, **C**_TX_, with shape (*n*, *n*_*x*_, *n*_*y*_).

This matrix is equal to the transmit field maps of all channels in arbitrary units. A normalization to obtain magnitudes of the relative $B_{1}^{+}$ maps in analogy to^11^ can be derived by

(4) $$\text{RelB}1{\mathrm{Mag}}_{i}=\frac{C_{\mathrm{TX},i}}{\sum \left|C_{\mathrm{TX},\mathrm{CP},i}\right|}.$$

Normalization in the above equations refers to circular polarized (CP) transmission using all channels so that **C**_TX,CP,*i*_ is given by

(5) $$C_{\mathrm{TX},\mathrm{CP},i}=C_{\mathrm{TX},i}\;e^{i\varphi_{\mathrm{CP}.i}},$$

with φ_CP,*i*_ containing the CP mode phase of the *i*‐th transmit array element, and the sum is taken over all transmit channels. Normalization of the transmit efficiency phase to that of the first array element, if desired, is achieved by

(6) $$\text{RelB}1{\text{Phase}}_{i}=\text{angle}\left(\frac{C_{\mathrm{TX},i}}{C_{\mathrm{TX},1}}\right).$$

To evaluate the proposed reconstruction method, relative $B_{1}^{+}$ maps were acquired using an 8TX/32RX coil array (Nova Medical) and an eight‐channel transceiver array. The second array uses off‐center fed dipoles arranged on a cylindrical former with a free diameter of 260 mm and is intended for use in a combined PET/MR system. $B_{1}^{+}$ maps for each of the arrays were acquired with the original method proposed by van de Moortele et al.^11^ and by the ESPIRiT techniques proposed here. For comparison, the approach proposed by Brunner et al.^19^ was used to derive maps from the same input data. GRE images were acquired when transmitting with each channel individually, using the following parameters: FA = 10°, TE = 4 ms, TR = 200 ms, matrix size = 128 × 128, averages = 1 and a total duration = 27 s with the shortest TR according to the range suggested in Cloos et al.^20^ Both phantom and in vivo images were acquired from the 8TX/32RX coil array. Because the PET/MR array has not yet been approved for in vivo measurements, it was only used to acquire phantom images. The phantom used was a cylindrical water phantom (diameter: 170 mm) doped with 50 mmol NaCl. All measurements were carried out in a 7 T TERRA system (Siemens Healthineers) equipped with eight transmit channels. In vivo images were acquired after approval of the study by the local ethics committee and after having obtained written informed consent from the volunteer subjects.

The calibration to the absolute maps used data acquired with a 3D AFI method acquisition with the following parameters: FA = 68°, TE1/TE2/TE3 = 2.04/6.12/10.20 ms, TR2 = 1000 ms, TR2/TR1 = 5, matrix size = 64 × 64, 16 slices, slice thickness = 4 mm, averages = 1, slices = 16, and a total duration = 9 min 51 s. The used sequence enabled simultaneous $B_{1}^{+}$ and ΔB_0_ mapping,^21^, ^22^ where the static field map was used to correct the flip angle maps.^23^ The AFI images were acquired when transmitting in CP mode, with all TX elements active, and only the slice located at the position identical to the GRE images was considered. Combining the relative $B_{1}^{+}$ maps of all channels linearly with the appropriate phases to achieve CP allows the computation of a scalar scaling factor that is valid for all voxels in contrast to other techniques^24^ where each voxel needs to be normalized individually. This scale factor was subsequently used to convert relative $B_{1}^{+}$ maps into absolute ones.

The wavelet‐based approach described in Donoho and Johnstone^25^ computes very high SNR values for the ESPIRiT‐based maps, therefore, restricting its applicability to Gaussian distributions only. SNR comparisons can also be based on statistical measures, like the Kullback–Leibler‐divergence (KL‐divergence),^26^ which measures the relative difference between two probability distributions. However, the singular value deomposition (SVD)‐based maps show a slightly different distribution to those computed with the original and the novel method, which renders KL‐divergence unsuitable in this case. This is why we compare the SNR of the $B_{1}^{+}$ maps generated with the different algorithms using the autocorrelation methods described in Sim et al.^27^ It should be noted that the SNR values obtained with this method depend on the way the images are cropped. Therefore, we refer to the SNR values obtained in this way as relative SNR, and all calculations are based on consistently cropping the outer 10 pixels.

The dynamic range of the proposed $B_{1}^{+}$ mapping was evaluated using simulations. For this purpose, the $B_{1}^{+}$ and $B_{1}^{-}$ maps of the PET/MR array were simulated in 3D electromagnetic simulations using the finite element method as implemented in the frequency domain solver of CST Microwave Studio (Dassault Systems, Vélizy‐Villacoublay, France). The field maps were fed into a Bloch equation simulator (JEMRIS^28^) where AFI and GRE images were simulated for a concentric two‐sphere phantom with T_1_ = 1000 ms/2000 ms and T_2_* = 25 ms/30 ms. The overall simulation workflow is described in more detail in Horneff et al.^29^

## 3 RESULTS

**FIGURE 1:**
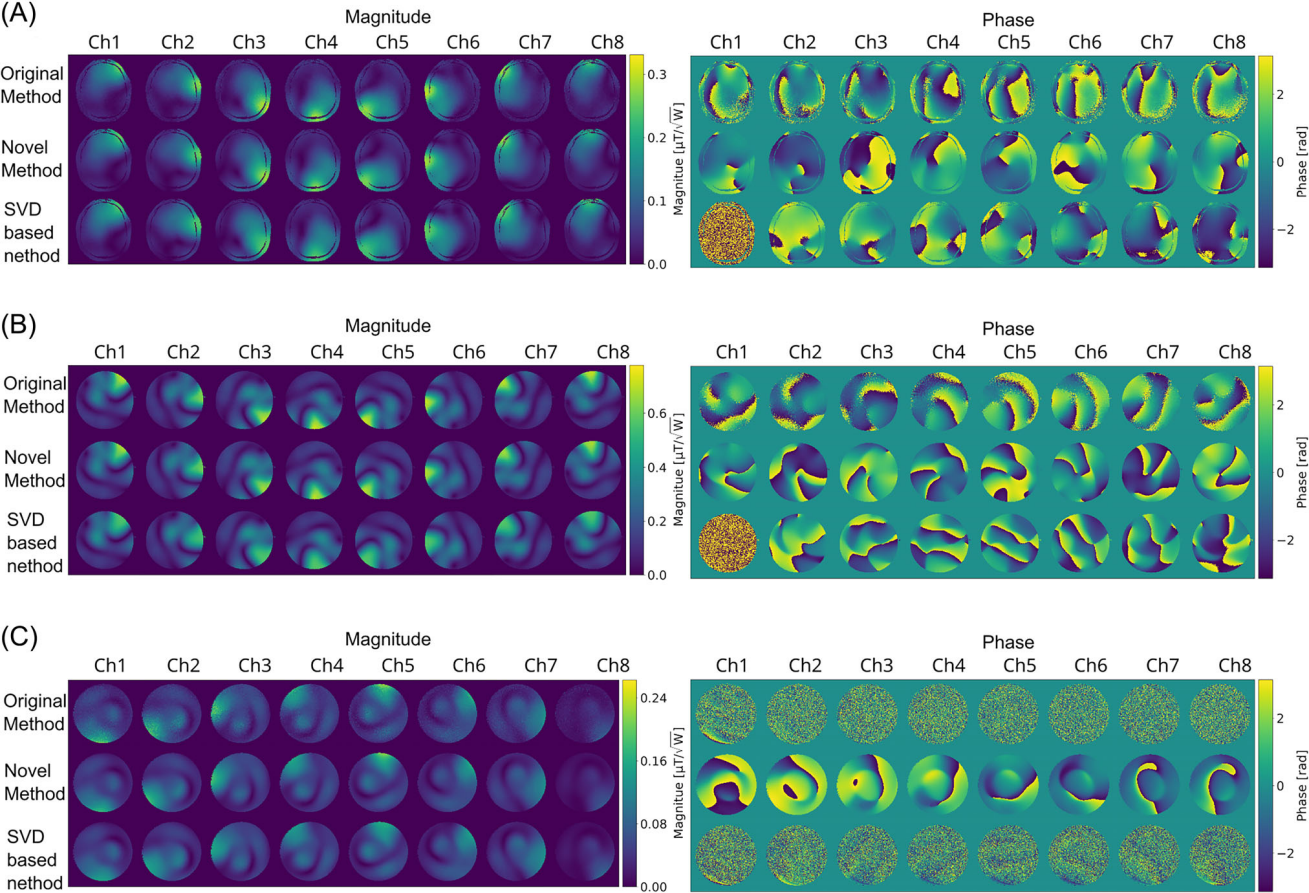
Absolute $B_{1}^{+}$ maps and unreferenced phase maps obtained with the method described by van de Moortele et al.,^11^ using the ESPIRiT‐based reconstruction and the singular value deomposition (SVD) approach described by Brunner et al.:^19^ (A) for the Nova 8TX/32 array in vivo (A) and in a phantom (B) and for the PET/MR array prototype (C). All maps are subjected to threshold masking to remove background noise.

Figure 1 shows absolute $B_{1}^{+}$ maps and corresponding unreferenced phase maps acquired with the reconstruction described in van de Moortele et al.^11^ These maps were obtained from the same measurement, but were reconstructed with the ESPIRiT algorithm and the SVD‐based reconstruction method described by Brunner et al.^19^ for two different RF coil arrays. An SNR gain in the magnitude maps created using the ESPIRiT reconstruction is visible compared to those obtained with the original method and, to a lesser extent, compared to those created with the SVD‐based approach. It can further be seen that exploitation of spatial smoothness through the application of a noise‐dominated null‐space within the ESPIRiT reconstruction creates higher SNR and smoother phase maps. The consistency of the phase maps is demonstrated in Figure **S1** where the phases have been normalized to the first array elements according to Eq. (6).

**FIGURE 2:**
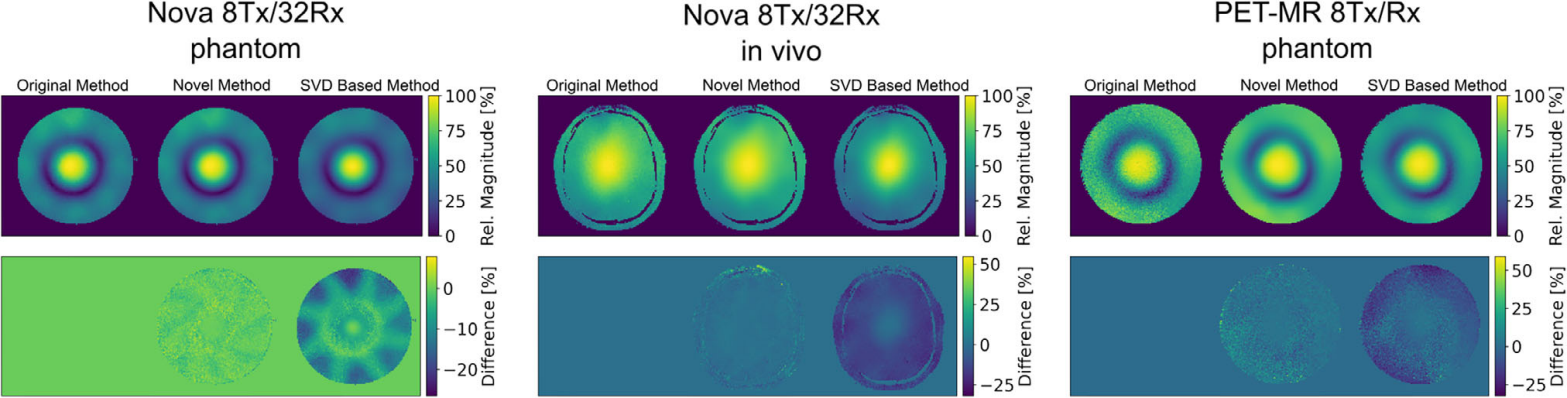
(First row) Circular polarized (CP) mode relative $B_{1}^{+}$ maps reconstructed with the method proposed by van de Moortele et al.,^11^ the modified method based on the ESPIRiT algorithm presented here and the singular value deomposition (SVD)‐based method described by Brunner et al.^19^ (Second row) Difference maps with respect to the original method of the CP mode maps shown above. All maps were subjected to threshold masking to remove background noise.

The SNR gain of the magnitude maps also holds for the CP mode relative $B_{1}^{+}$ maps generated by linear superposition, as described above and shown in the first row of Figure 2. In addition, difference images from the CP mode maps are given in the second row of Figure 2. It can be seen that the SVD‐based approach creates systematic deviations from the original method, whereas the difference image from our novel method mostly displays removed noise. The quantitative comparison of the relative maps shown in Figure 2 is given in Table 1 as the relative SNR based on the autocorrelation method. Again, it can be seen that our novel method consistently provides higher SNR by a factor of 3 to 4 for all SNR estimators given in Sim et al.^27^ compared to the methods proposed by van de Moortele et al.^11^ and Brunner et al.^19^ and achieves an infinitely larger SNR for the National Electrical Manufacturers Association (NEMA) standard method. The SVD based method described by Brunner et al.^19^ produces a lower SNR for all SNR estimators given in Sim et al.,^27^ compared against the original method described by van der Moortele et al.^11^

**TABLE 1:** Comparison of the quality of the relative $B_{1}^{+}$ maps obtained with the three reconstruction methods for different coils and objects being imaged.

	Relative SNR original method	Relative SNR novel method	Relative SNR SVD‐based method
Nova coil (phantom)	3.59–3.65 (2.75)	11.37–12.62 (∞)	2.51–2.56 (2.53)
Nova coil (in vivo)	2.6.78–6.90 (4.03)	15.35–16.10 (∞)	4.75–4.81 (3.76)
PET/MR coil (phantom)	5.11–5.25 (3.45)	4.93–5.10 (∞)	4.33–4.40 (3.63)

*Note*: The range of relative SNR given spans the range computed with the three SNR estimators given in Sim et al.^27^ The value in parentheses represents the SNR value computed according to the National Electrical Manufacturers Association standard method 4.

**FIGURE 3:**
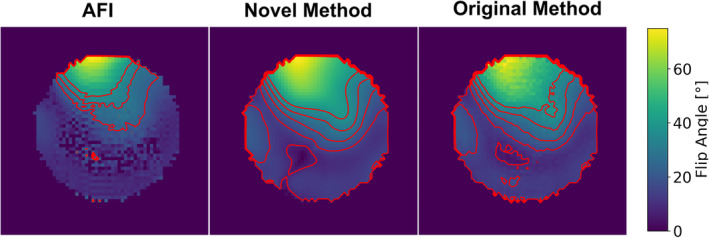
Simulated single element $B_{1}^{+}$ maps obtained with the AFI method (nominal flip angle [FA] = 75°) (left), the novel method based on ESPIRiT (middle), and the original method (right). The images show isocontours in red for 30%, 40%, and 50% of the maximum value for the AFI map and 10% to 50% in 10% steps for the other two maps. All three maps have been threshold‐masked to remove background noise.

Simulated $B_{1}^{+}$ maps for a single transmit element of the PET/MR array are shown in Figure 3. Compared with the directly computed map from the AFI simulation, both $B_{1}^{+}$ maps based on GRE images for the relative maps and calibration with a single CP mode AFI acquisition show a higher dynamic range—as can be seen by the larger range of isocontour lines in these maps. Direct comparison of the $B_{1}^{+}$ maps computed with the original method by van de Moortele and the ESPIRiT‐based reconstruction reveal the smoothing effect of the ESPIRiT algorithm. Although both maps are plotted for a matrix size of 64 × 64, the middle map shows smoother isocontour lines, which also contributes to the visual impression of a higher SNR. This is the case even though no noise was added to the k‐space data, which was simulated using the JEMRIS Bloch equation simulator.

## 4 DISCUSSION AND CONCLUSIONS

We have demonstrated the application of the ESPIRiT algorithm, widely used in parallel imaging reconstruction, for computing relative $B_{1}^{+}$ maps from a set of gradient echo images. Similar to the established method proposed by van de Moortele et al.,^11^ the ESPIRiT‐based reconstruction normalizes the relative transmit sensitivity maps to the CP‐mode. This normalization enables the efficient generation of absolute sensitivity maps using a single absolute flip‐angle measurement in CP‐mode, for instance, via the AFI sequence.

However, unlike the method by van de Moortele et al.,^11^ the ESPIRiT‐based approach significantly enhances the SNR in the transmit $B_{1}^{+}$ fields. This improvement is attributed to the ESPIRiT model's ability to account for both intra‐ and inter‐voxel relationships, whereas the van de Moortele method considers only intra‐voxel relationships, treating each voxel independently.

When comparing the ESPIRiT‐based approach to the SVD‐based algorithm suggested by Brunner et al.^19^, we observe superior noise suppression with ESPIRiT. This advantage is likely because of the fact that similarly to van de Moortele's method, Brunner's method does not consider inter‐voxel relationships. More importantly, we found that the method proposed by Brunner et al.^19^ systematically deviates from the results of van de Moortele et al.,^11^ whereas the ESPIRiT method does not. This discrepancy may arise from the way in which Brunner's method normalizes the relative sensitivity maps—ensuring that the TX/RX matrices are voxelwise unitary—whereas both the van de Moortele method and our ESPIRiT‐based approach normalize voxelwise to the physics‐based CP‐mode, defined as the voxelwise sum of the channel magnitudes.

Improved SNR using this technique is highly beneficial as it enables fast acquisition of high dynamic range $B_{1}^{+}$ maps. The lower SNR of the previous implementation often required the use of signal averages to obtain high‐quality maps in both phantom (e.g., Zhang et al.^16^) and in vivo measurements (e.g., Wu et al.^15^). In contrast, high‐quality maps could be obtained with the ESPIRiT technique without averaging, as demonstrated for multiple coil arrays in Figure 1. The gain in SNR is especially useful when evaluating transmit arrays during construction when no multi‐channel RX array is present. In this case, the ESPIRiT‐based generation of $B_{1}^{+}$ achieves the highest SNR gain in the phantom measurements. Because highly optimized open‐source implementations of ESPIRiT are available (e.g., in the BART toolbox) our proposed approach can be readily adopted. The fast implementation also reduced the computation time, which is critical in pTX applications, because preparation time to estimate the transmit sensitivity maps is a limiting factor in widespread adoption.

It should be noted that interferometric acquisitions of $B_{1}^{+}$ maps, as mentioned in the introduction, promise a higher dynamic range then measurements obtained when transmitting with a single array element. Simulated maps for this case was derived in an identical way to the one used to generate Figure 3 and are shown in Figure S2. It can be seen that the dynamic range obtained in this way is increased compared to when transmitting with a single element, but does not reach the range of the original or novel GRE‐based mapping method. Figure S3 shows the influence of varying the nominal flip angle of the AFI sequence on the dynamic range of the computed maps. Again, it can be seen that the GRE‐based methods retain higher fidelity.

Although our implementation used the relatively slow but accurate AFI method to acquire a CP mode flip angle map, it is worth noting that a faster mapping sequence, such as DREAM,^4^ has previously been used for this purpose in Beqiri et al.^30^ In this case, a total acquisition time (TA) in the range of TA = 2 min for an eight‐channel TX array and a matrix size of 64 × 64 can be realized. Speed enhancement, including the absence of averaging and faster absolute mapping, is essential when using the described mapping method as input for B_1_‐shimming or parallel‐transmit pulse calculation.^20^, ^31^ A combination of the ESPIRiT‐based reconstruction of $B_{1}^{+}$ maps in conjunction with a fast DREAM acquisition might also be useful in body applications where SAR does often not limit the achievable flip angle, but rather, the available transmit power is the limiting factor. Therefore, the low FA gradient echoes in combination with a fast DREAM or time‐interleaved acquisition of modes^24^‐based acquisition of absolute maps in CP mode present promising avenues.

Finally, normalization of $B_{1}^{+}$ phase maps to an arbitrary channel may result in errors in parallel transmit pulse design (e.g., as reported in Krueger et al.^32^) This is why normalization is often carried out with respect to two virtual coil elements. The smoothness of the phase maps obtained using the ESPIRiT reconstruction without normalization might be sufficient to avoid the normalization step before pulse computation. This will be investigated in the future.

## CONFLICT OF INTEREST STATEMENT

N.J.S. and J.F. are co‐founders of Affinity Imaging a spin‐off company that manufactures high field MRI coils for research purposes.

## Supporting information


**Figure S1.** Figure 1 of the manuscript repeated but with phases normalized to that of channel #1, thus showing the consistency of the phases obtained with the different reconstruction algorithms.
**Figure S2.** Simulated $B_{1}^{+}$ maps of the PET/MR array using AFI in all‐but‐one encoding. (top row): raw maps and (bottom row) maps decomposed for individual transmit channels.
**Figure S3.** Simulated $B_{1}^{+}$ maps of the PET/MR array with varying nominal flip angles of 50° (top row), 75° (middle row) and 90° (bottom row). The columns show, from right to left: AFI acquisition with a single transmit channel, AFI acquisition obtained interferometrically, novel method based on the ESPIRiT algorithm and original method.
