# Supplementary material for: High dynamic range B1+ mapping for the evaluation of parallel transmit arrays
Source: Magn Reson Med. 2024 Oct 27;93(3):1298–305. doi: 10.1002/mrm.30349 (PMC11680734; doi:10.1002/mrm.30349)
Supplement: Supplementary file 1 — Figure S1. Figure 1 of the manuscript repeated but with phases normalized to that of channel #1, thus showing the consistency of the phases obtained with the different reconstruction algorithms. Figure S2. Simulated B1+ maps of the PET/MR array using AFI in all‐but‐one encoding. (top row): raw maps and (bottom row) maps decomposed for individual transmit channels. Figure S3. Simulated B1+ maps of the PET/MR array with varying nominal flip angles of 50° (top row), 75° (middle row) and 90° (bottom row). The columns show, from right to left: AFI acquisition with a single transmit channel, AFI acquisition obtained interferometrically, novel method based on the ESPIRiT algorithm and original method. [file MRM-93-1298-s001.docx]

Supplementary Material

**Normalized Phase Maps**

**
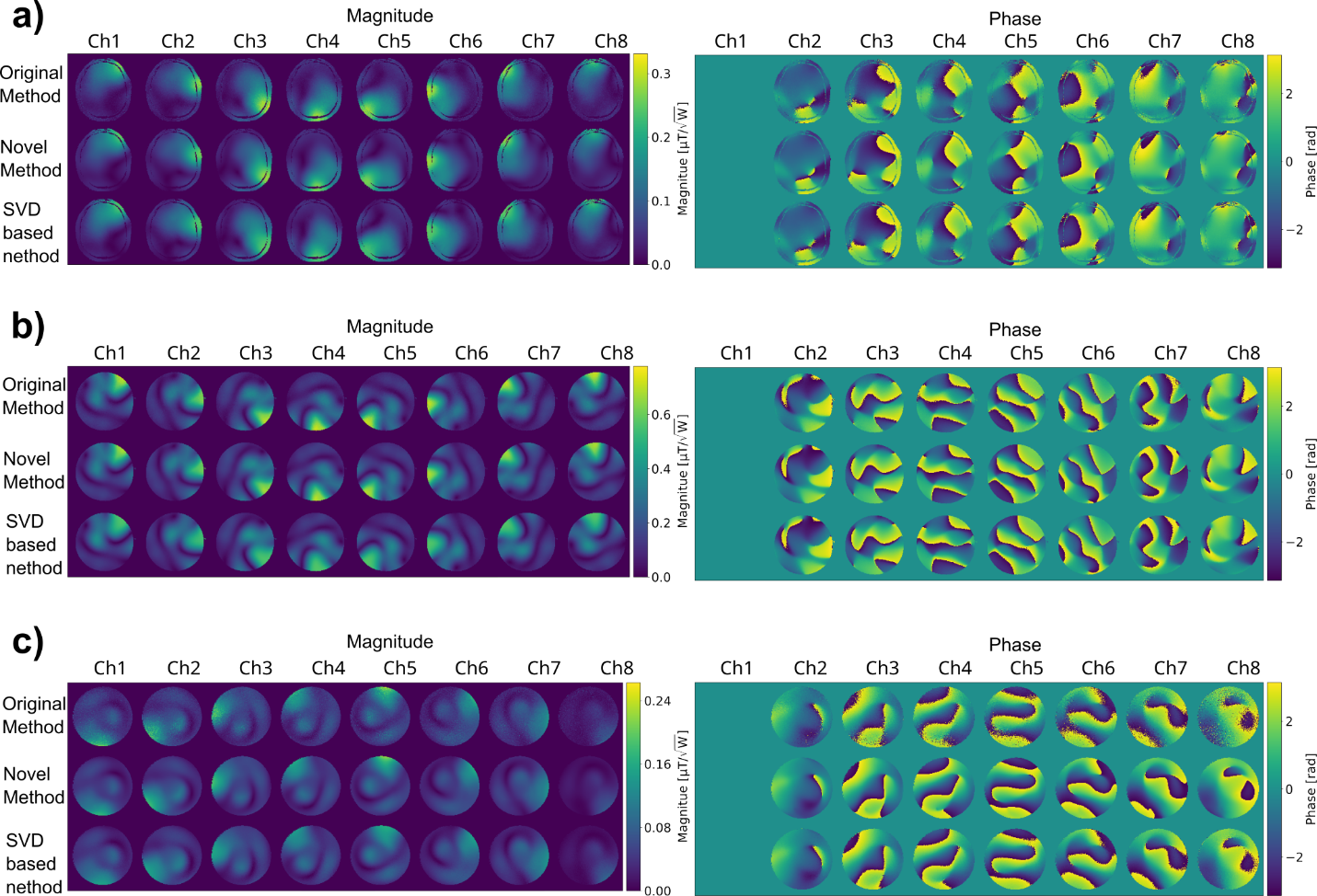
**

Figure S1: Figure 1 of the manuscript repeated but with phases normalized to that of channel #1, thus showing the consistency of the phases obtained with the different reconstruction algorithms.

**Interferometric AFI**


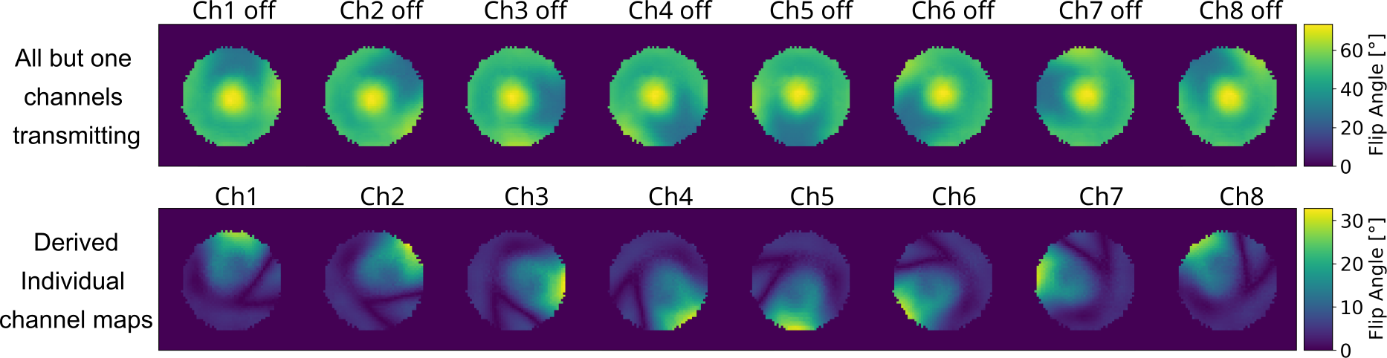


Figure S2: Simulated B_1_^+^ maps of the PET/MR array using AFI in all-but-one encoding. (top row): raw maps and (bottom row) maps decomposed for individual transmit channels.

**Dynamic range versus nominal flip angle**

**
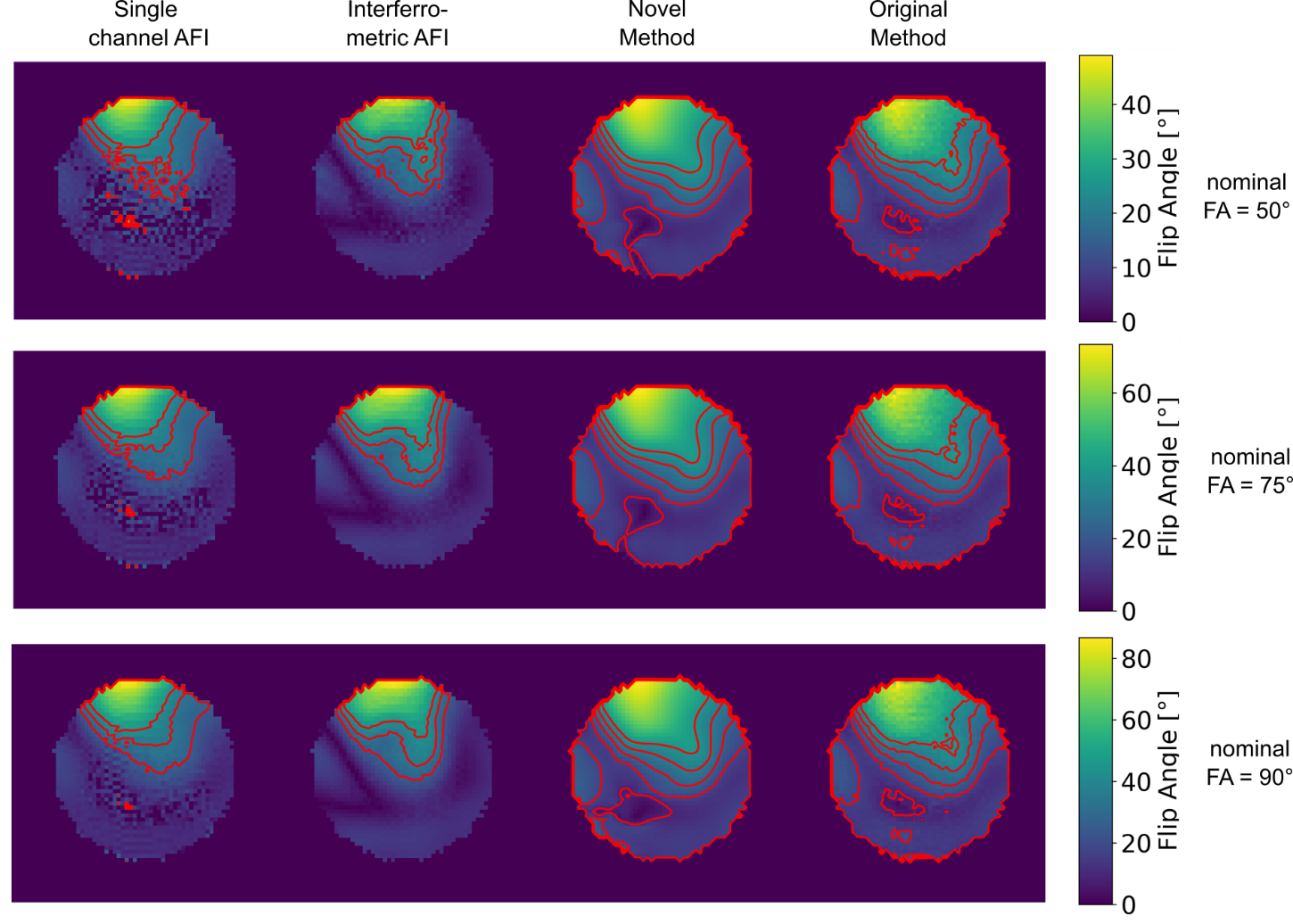
**

Figure S3: Simulated B1+ maps of the PET/MR array with varying nominal flip angles of 50° (top row), 75° (middle row) and 90° (bottom row). The columns show, from right to left: AFI acquisition with a single transmit channel, AFI acquisition obtained interferometrically, novel method based on the ESPIRiT algorithm and original method.
